# Controversy Over Bullets and in the Bully Pulpit

**DOI:** 10.1100/tsw.2001.2

**Published:** 2001-01-12

## Abstract

Nature leads with a story about the suspected double-whammy packed by bullets made of depleted uranium. A slew of new appointees in both houses of the U.S. Congress leads the news in Science this week.

Can the radioactive bullets used by NATO in the 1999 Kosovo conflict kill in two ways? Not likely, say radiation experts in *Nature's* lead story. *Nature* reports that a "fierce political debate" has erupted in Europe this month after six Italian soldiers who served in the conflict were diagnosed with leukemia. The governments of Italy, Belgium, Portugal and Sweden have each targeted a surprising culprit: uranium bullets. Uranium-238, a mildly radioactive by-product of nuclear power production, is prized by bullet-makers for its high density. Nearly eight tonnes of the (faintly) glowing metal fell on Serbian targets during the 1999 conflict in Kosovo, reports *Nature*. That is a lot of uranium, but far too little to cause the large-scale radioactive contamination necessary to cause a cancer epidemic, conclude scientists interviewed by *Nature*. Instead, the scientists point to typical wartime misuse of carcinogenic lubricants, solvents and disinfectants, says *Nature*. TWINS appreciates this argument. We'd be in a hurry (and probably splash a little grease) too, if we were working under a rain of radioactive shrapnel. *Science* did not cover this story.

*Science* leads this week with an article evaluating several new appointees to key science policy positions in Washington. The warmest reception, according to policy specialists interviewed by *Science*, will go to the new leader of the Science Committee in the House of Representatives, Sherwood Boehlert of New York. Although Boehlert is a Republican, he is generally seen as a "liberal" member of his party, says *Science*. The magazine reports that Boehlert's staff director, David Goldston, believes that his boss will focus on topics more familiar to Democrats: math and science education, alternative energy sources, and environmental research. It sounds optimistic, but TWINS notes that the sword of "focus" cuts both ways. At least Boehlert is paying attention. The same can't be said for Christine Todd Whitman. *Science* reports that President-elect George Bush's pick to lead the Environmental Protection Agency recently worried climate scientists by confusing the causes of ozone depletion and climate change. Other less familiar choices reported by the magazine include Ralph Regula, the new head of the House appropriations subcommittee that oversees the National Institutes of Health (NIH's) budget, Sonny Callahan, head of the House spending panel that oversees the Department of Energy's budget, and Arlen Specter, who will continue to chair the Senate committee that oversees the NIH.

*Nature* didn't comment on any of these appointees, but did note in a separate article this week that the Bush Administration's decision to nominate Donald Rumsfeld for Secretary of Defense could indicate a revived interest in developing a space-based missile defense program. Rumsfeld is a long-time proponent of missile defense and recently headed a congressional commission that endorsed "US control of space, including defending our own satellites and engaging those of any enemy." Space policy specialists interviewed by *Nature* did not endorse Rumsfeld.
